# Structure and evolution of double minutes in diagnosis and relapse brain tumors

**DOI:** 10.1007/s00401-018-1912-1

**Published:** 2018-09-28

**Authors:** Ke Xu, Liang Ding, Ti-Cheng Chang, Ying Shao, Jason Chiang, Heather Mulder, Shuoguo Wang, Tim I. Shaw, Ji Wen, Laura Hover, Clay McLeod, Yong-Dong Wang, John Easton, Michael Rusch, James Dalton, James R. Downing, David W. Ellison, Jinghui Zhang, Suzanne J. Baker, Gang Wu

**Affiliations:** 1 Department of Computational Biology, St. Jude Children’s Research Hospital, MS1135, 262 Danny Thomas Place, Memphis, TN 38105 USA; 2 Department of Pathology, St. Jude Children’s Research Hospital, 262 Danny Thomas Pl, Memphis, TN 38105 USA; 3 Department of Developmental Neurobiology, St. Jude Children’s Research Hospital, 262 Danny Thomas Pl, Memphis, TN 38105 USA

**Keywords:** Clonal evolution, Copy number alteration, Double minutes, Structural variation, Tumor heterogeneity

## Abstract

Double minute chromosomes are extrachromosomal circular DNA fragments frequently found in brain tumors. To understand their evolution, we characterized the double minutes in paired diagnosis and relapse tumors from a pediatric high-grade glioma and four adult glioblastoma patients. We determined the full structures of the major double minutes using a novel approach combining multiple types of supporting genomic evidence. Among the double minutes identified in the pediatric patient, only one carrying *EGFR* was maintained at high abundance in both samples, whereas two others were present in only trace amounts at diagnosis but abundant at relapse, and the rest were found either in the relapse sample only or in the diagnosis sample only. For the *EGFR*-carrying double minutes, we found a secondary somatic deletion in all copies at relapse, after erlotinib treatment. However, the somatic mutation was present at very low frequency at diagnosis, suggesting potential resistance to the *EGFR* inhibitor. This mutation caused an in-frame RNA transcript to skip exon 16, a novel transcript isoform absent in EST database, as well as about 700 RNA-seq of normal brains that we reviewed. We observed similar patterns involving longitudinal copy number shift of double minutes in another four pairs (diagnosis/relapse) of adult glioblastoma. Overall, in three of five paired tumor samples, we found that although the same oncogenes were amplified at diagnosis and relapse, they were amplified on different double minutes. Our results suggest that double minutes readily evolve, increasing tumor heterogeneity rapidly. Understanding patterns of double minute evolution can shed light on future therapeutic solutions to brain tumors carrying such variants.

**Electronic supplementary material:**

The online version of this article (10.1007/s00401-018-1912-1) contains supplementary material, which is available to authorized users.

## Introduction

Double minutes are extrachromosomal circular DNA (eccDNA) frequently found in many different tumor types, especially in brain tumors [2, 3, 34]. Double minutes are often found in the context of chromothripsis, a catastrophic event in which one or a few chromosomes are shattered into pieces, then reassembled in an unnatural order and orientation through non-homologous end joining and/or microhomology-mediated break repair [15, 27]. Due to their lack of centromeres, double minutes are unequally segregated to daughter cells at cell division. Recent mathematical modeling shows that eccDNA amplification can increase copy number of oncogenes more effectively compared to chromosomal amplification; consequently, it would significantly enhance and maintain tumor heterogeneity [32]. Double minutes in tumor cells have been found to confer resistance to targeted cancer therapy [19, 26]. Recently it has been shown that cell-free eccDNA can be detected in plasma or serum and is possibly more stable than circulating linear DNA [12]. Despite their important roles in tumorigenesis and clinical utilities, it is not clear how double minutes evolve and how they contribute to the dynamics of tumor heterogeneity.

With the availability of high-depth whole genome sequencing (WGS) of the paired tumor and normal samples from the same patient, it is possible to assemble the circular structure of double minutes based on short-read sequencing data. For instance, Sanborn et al. [22] published a method to determine full structures of double minutes using WGS data from The Cancer Genome Atlas (TCGA) glioblastoma multiforme (GBM) samples. More recently, AmpliconArchitect was developed for searching and constructing circular DNA structures based on discordant reads [32]. Recent studies have also reported the structural heterogeneity of double minutes in a tumor population in several cancer types, suggesting that they dynamically evolve [13, 14]. In this study, we sought to examine the evolution of double minutes in details in a set of paired diagnosis and relapse tumors from a pediatric high-grade glioma (HGG) patient. We constructed the fine structures of the double minutes in each sample using short read (Illumina sequencing) data followed by validation with long read (Chromium linked-reads sequencing) data. Specifically, the double minute structures were inferred from cyclic graphs in a network built based on highly amplified somatic copy number alteration (CNA) segments and structural variants (SVs) associated with the segment boundaries. We estimated the copy number of each double minute based on the allele frequencies of the single nucleotide variants (SNVs) present in the pertinent CNA segments. Our results reconstructed the dynamics of double minute evolution from diagnosis to relapse in this patient. We provided evidence that secondary somatic mutations on a double minute can drastically affect its fitness as revealed by copy number in the relapse tumor compared to diagnosis.

We analyzed four additional pairs of diagnosis and relapse samples from TCGA GBM patients and observed similar dynamics of copy number shift of double minutes between the diagnosis and relapse tumors. We generalized an evolutionary model to account for the observations made in these five sets of longitudinal data. The model suggests that double minutes follow an evolutionary trajectory independent of that of linear chromosomes.

## Methods

### Sequencing data of matched diagnosis-relapse-germline samples

Patient SJHGG019 was treated on St. Jude Children’s Research Hospital protocol (NCT00124657), with informed consent, and this study was approved by the institutional review board at St. Jude Children’s Research Hospital. The matched diagnosis, relapse, and germline samples from SJHGG019 were previously described in Wu et al. [36]. All samples were subject to Illumina paired-end WGS with 100 bp read length. The two tumor samples have 57× average coverage and the germline sample has 36× average coverage. The bam files for this patient are available at European Bioinformatics Institute, under accession EGAS00001000192.

Another four sets of WGS data were downloaded from the National Cancer Institute’s Genomic Data Commons Legacy Archive with the following case IDs: TCGA-06-0125, TCGA-06-0152, TCGA-06-0211, and TCGA-14-1402 [8]. Specifically, G49538.TCGA-06-0125-01A-01D-1490-08.2.bam (diagnosis), G49538.TCGA-06-0125-02A-11D-2280-08.2.bam (relapse), and G49538.TCGA-06-0125-10A-01D-1490-08.2.bam (germline) were downloaded for the TCGA-06-0125 case. G2145.TCGA-06-0152-01A-02D.12.bam (diagnosis), G49538.TCGA-06-0152-02A-01D-2280-08.1.bam (relapse), and G2145.TCGA-06-0152-10A-01D.13.bam (germline) were downloaded for the TCGA-06-0152 case. G49538.TCGA-06-0211-01A-01D-1491-08.1.bam (diagnosis), G49538.TCGA-06-0211-02A-02D-2280-08.1.bam (relapse), and G49538.TCGA-06-0211-10A-01D-1491-08.1.bam (germline) were downloaded for the TCGA-06-0211 case. G49538.TCGA-14-1402-01A-01D-1493-08.3.bam (diagnosis), G49538.TCGA-14-1402-02A-01D-2280-08.3.bam (relapse), and G49538.TCGA-14-1402-10A-01D-1493-08.3.bam (germline) were downloaded for the TCGA-14-1402 case.

### Reconstructing the structures of double minutes

#### Extracting highly amplified regions

For each tumor sample, we ran CONSERTING on paired tumor-germline samples to detect CNA segments [4]. The magnitude of the CNA segments is measured by log_2_ ratio (log_2_R) of the coverage signal between tumor and paired germline sample. A log_2_R greater than zero means copy number gain and a log_2_R smaller than zero means copy number loss. Since we are interested in constructing structures of highly amplified double minutes, we extracted CNA segments that fall in the right tail of the empirical log_2_R distribution (approximately two standard deviation above mean) as the highly amplified CNA segments. In a few TCGA samples, we manually adjusted the extracted segments’ boundaries if they are not very consistent with the sequencing coverage signal viewed on IGV 2.3.91 [31].

#### Identifying SVs around the boundaries of each segment

For each segment, we pulled the reads mapped to the [− 50 bp, 50 bp] flanking regions of its 5′ boundary and its 3′ boundary using SAMtools [16]. If a read is soft-clipped, i.e., its CIGAR column contains both “S” and “M”, we BLAT the read against the reference genome GRCh37 [11]. We then searched whether their BLAT hits are uniquely mapped to the [− 1 kb, 1 kb] flanking regions of any one of the other segments’ boundaries. If so, we counted the read once for “soft-clipped read” evidence of SV between one boundary and another boundary. The signs and mapped positions of the read indicate the orientations of the two fused segments.

For the reads mapped to the [− 50 bp, 50 bp] flanking region of each segment boundary, we also extracted the reads that have their paired reads mapped to either another chromosome or the same chromosome but with a distance greater than 800 bp as discordant read pairs. We then checked whether their discordantly mapped paired reads are in the [− 1 kb, 1 kb] flanking regions of any one of the other segments’ boundaries. If so, we counted the discordant read pair once for “discordant reads” evidence of SV between one segment boundary and another segment boundary. The FLAG column in the BAM file indicates the orientations of the two linked segments. The SVs supported by discordant reads but not soft-clipped reads are due to repetitive or unknown sequences between the two linked segments.

Finally, we checked whether the discordantly mapped paired reads of the reads at the [− 50 bp, 50 bp] flanking regions of any two segments’ boundaries are within a distance of 10 kb and whether their orientations are consistent, which may suggest that between the two potentially linked segments is a non-repetitive sequence, such as a small CNA segment missed by CONSERTING, flanked by repetitive/unknown sequences. If so, we counted such case once for “bridging discordant reads” evidence of SV between one segment boundary and another segment boundary.

The above mentioned parameters such as the 50-bp flanking region, 1-kb distance to segment boundaries, and 10-kb distance in the bridging discordant reads call can all be adjusted to accommodate different samples. For example, in the TCGA-06-0152 samples, we used 100 bp instead of 50 bp flanking region and we used 3 kb instead of 1 kb distance to segment boundaries.

For SVs identified by any of the above three scenarios, we manually checked their BLAT results and the mapped reads in SAM format to make sure the identified SVs are true positives.

#### Constructing network and identifying cyclic graphs

For each sample, we built a bidirected graph with each node representing a boundary of a CNA segment and edges including: (1) segment edges, i.e., the CNA segments; (2) SV edges, i.e., the identified SVs connecting the boundaries of two segments; and (3) adjacent edges, i.e., edges connecting two segments that are next to each other on the reference genome.

We enumerated all simple cycles of the constructed graph using Johnson’s algorithm [10]. As the graph is bidirectional, each simple cycle would appear twice with opposite directions, representing two strands with opposite orientations in a double minutes. We first preprocessed the simple cycles by removing one of the two cycles with the same set of nodes and edges but opposite directions. A side effect of using two nodes instead of one node to represent a segment in the graph is that some simple cycles may not be valid as only one boundary of a segment may be included in the cycle. We, therefore, further removed such simple cycles to form the final candidates set.

The codes, supporting data and detailed documentation of the processes have been made available at https://github.com/stjude/Episomizer.

### Chromium sequencing validation

To validate the double minute structures inferred from short-read sequencing data, we performed linked-read sequencing for the diagnosis and relapse tumor samples of SJHGG019 using Chromium WGS platform by 10X Genomics^®^. Briefly, 1 ng genomic DNA was input, each molecule was captured by a gel bead which contains specific barcode to distinguish different molecules on 10X Genomics Chromium Controller, then amplified by isothermal incubation. The barcoded DNA was purified, then end repaired, dA-tailed and adaptor ligated using chromium genome library kit. Finally, the library was purified and enriched by index PCR amplification then sequenced paired-end 151 cycles on Illumina HiSeq 4000 [39]. The sequencing data were processed using the Long Ranger pipelines (version 2.1.5) and visualized by Loupe (version 2.1.2). For the diagnosis tumor sample, the average molecule length is 26 kb; and for the relapse tumor sample, the average molecule length is 31 kb. The bam files of the Chromium data are available at European Bioinformatics Institute, under accession EGAS00001003212.

### Tri-color FISH analysis

The FISH probes used in this study are all from BACPAC Resources (Oakland, CA): *CDK6* (RP11-888H2/CH17-182D03, 7q21.2, SpectrumAqua), *EGFR* (RP11-148P17/RP11-1083E20, 7p11.2, SpectrumGreen) and *C*-*MYC* (CTD-3056O22/CTD-2267H22, 8q24, Rhodamine). The probes were applied to de-paraffinized tissue samples. The samples and probes were co-denatured at 90 °C for 12 min, hybridized overnight at 37 °C, washed in a 50% formamide solution for 5 min at 25 °C, and then stained with a DAPI counterstain. Signals were reviewed using a fluorescent microscope equipped with appropriate filters [7].

### Germline and somatic SNV call

The germline variants were called using bambino [6]. BLAT search was used to retain high-quality SNVs uniquely mapped to only one genomic location [11]. Somatic mutations were determined as previously described [36]. Variant allele frequency (VAF) was calculated as Alternative allele read count/Coverage depth, where Coverage depth = Reference allele read count + Alternative allele read count.

### RNA-seq analysis

The RNA-seq data were mapped using StrongArm as previously described [36]. Due to the lack of RNA from the diagnosis sample of SJHGG019, the RNA-seq data are only available for the relapse tumor of SJHGG019. The read count per gene was determined by HT-Seq Count and converted to FPKM [1]. RNA-seq data at *EGFR* locus for 11,094 TCGA samples were downloaded from NCI GDC data portal using “*bamslicing”* function. The splicing reads spanning exon junctions were quantified by RNApeg [Edmonson et al. in prep]. Additional RNA-seq data were from pediatric non-brainstem HGGs [36], and from non-tumor tissues including 683 brain tissues (33 from [38]; 650 from HDBR [17]) and 95 samples of other tissue types (myeloid, *n* = 9; prostate, *n* = 15; kidney, *n* = 7; cord blood, *n* = 52; bone marrow, *n* = 12). Statistical analysis and plotting of the RNA-seq data were conducted in Rstudio with R version 3.3.2 [30].

## Results

### Structures of double minutes in the relapse tumor of SJHGG019

**Fig. 1:**
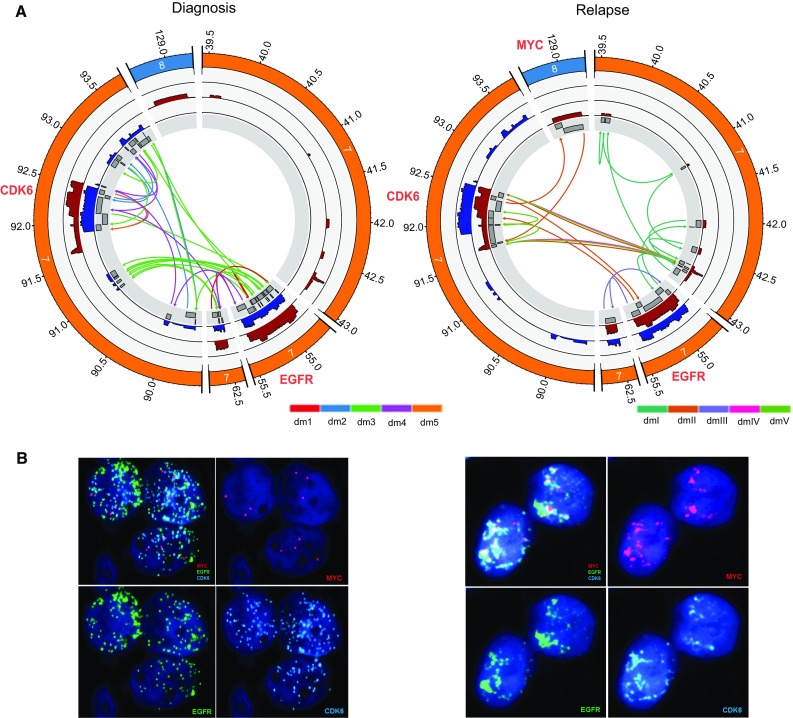
Predicted structures of double minutes and FISH analysis in the diagnosis and relapse samples of SJHGG019. **a** CIRCOS plots from the most inner circle to the most outer circle represent the highly amplified genomic segments, sequencing coverage of the diagnosis/relapse sample (blue/maroon) and genomic coordinates (×1000 kb) of the chromosomes. SVs between the segment boundaries are shown as arrows indicating the orientations of the joined segments constituting a double minutes. For each sample, the SVs associated with different double minute structures are colored differently. **b** Tri-Color FISH experiment shows that *EGFR* (green) in both samples, *CDK6* (aqua) in both samples, *MYC* (magenta) in the relapse sample only, are localized to multiple punctae that are widely distributed in the nucleus of representative cells, suggesting that the presence of different double minutes. For each sample, the four panels represent FISH results for all three genes together and each gene separately

SJHGG019 is from a 7-year-old male patient, diagnosed with GBM (WHO grade IV) in the right cerebellar hemisphere. He was treated in a clinical trial of an *EGFR* inhibitor, erlotinib (tarceva) [21]. Recurrent anaplastic astrocytoma (WHO grade III) was found in the same anatomical region after 11 months. The patient succumbed to his disease 2 months later. The complete profiles of somatic mutations in the diagnosis and relapse tumors were reported in a larger study to depict the landscape of the somatic mutations in pediatric HGGs [36]. Here we further examined the evolutionary dynamics of double minutes in the tumors of this patient. Copy number analysis of the WGS data from matched relapse-germline samples of the patient identified 531 genomic segments with a log_2_ ratio (log_2_R, the coverage signal between tumor and paired germline samples) ranging from − 0.28 to 6.61 (Suppl. Figure 1, Online Resource 1; Suppl. Table 1, Online Resource 2). The segments with log_2_R values falling on the far right tail of the distribution are highly amplified (Suppl. Figure 1, Online Resource 1). Using an empirical threshold of log_2_R > 4 to define the high copy number segments, 29 segments are retained. The majority of the segments are located on chromosome 7, with only two located on chromosome 8 (Fig. 1a, Suppl. Figure 2, Online Resource 1). Among the 29 segments, there are 18 pairs of adjacent segments, meaning that their genomic locations are next to each other but have fluctuated coverage depths or log_2_R (Suppl. Figure 2, Online Resource 1). Among the 58 boundaries of these 29 segments, we identified 15 SVs supported by evidence of both soft-clipped reads and discordant read pairs, one SV supported by discordant read pairs but not soft-clipped reads, and one SV supported by bridging discordant reads only (Fig. 1a, Suppl. Table 1, Online Resource 2; Suppl. Figures 3, 4, Online Resource 1; see “Methods” section for definition of “bridging discordant reads”). Further examination of this “bridge” SV reveals that their shared discordant reads are likely a tiny region (about ~ 150 bp) within segment 6 (seg6, Suppl. Table 1, Online Resource 2).

To confirm the structure and the orientation of the SVs detected, we performed an orthogonal sequencing approach on the same DNA sample with Chromium linked-reads WGS at 30X genome wide coverage depth. Every SV detected by our method was validated by Chromium linked-reads data. For example, the linked-reads data of the two SVs supported by discordant read pairs only and bridging discordant reads only, separately, were shown in Suppl. Figure 5, Online Resource 1, where the x- and y-axis represent the genomic locations of the two segments and the color intensity represent the number of shared barcodes between any two genomic coordinates. The darker the color is, the more barcodes the two segments share and thus the more likely they are from the same molecule. Notice that in the heatmaps, dark color in the lower right or upper left corners means →→ (head to tail) type of rearrangement. Dark color in the lower left corners means ←→ (tail to tail) type of rearrangement, and dark color in the upper right corner means →← (head to head) type of rearrangement.

If we treat every segment boundary as a node in a conceptual network, and the edges as one of the three following types: (1) segment edges (for the segment itself), (2) adjacent edges (for segments next to each other), and (3) SV edges, we find that for most of the 29 highly amplified segments, their boundaries can be connected to other segments’ boundaries through either SV or adjacent edges. The only exception is seg18′s 3′ end (18R) and seg13′s 5′ end (13L, dmIII, lower panel of Suppl. Figure 3, Online Resource 1). Neither of the two nodes connects to any segment boundaries. We, therefore, used Chromium linked-reads data to see whether a SV between these two dangling nodes exists. Interestingly, we found strong evidence of SV between 18R and 13L with these data (Suppl. Figure 5C, Online Resource 1). The reason that we identified the SV between 18R and 13L through Chromium linked-reads but not discordant read pairs or soft-clipped reads is likely because the length of the repetitive or unknown region between the two segments is longer than the insert size.

**Fig. 2:**
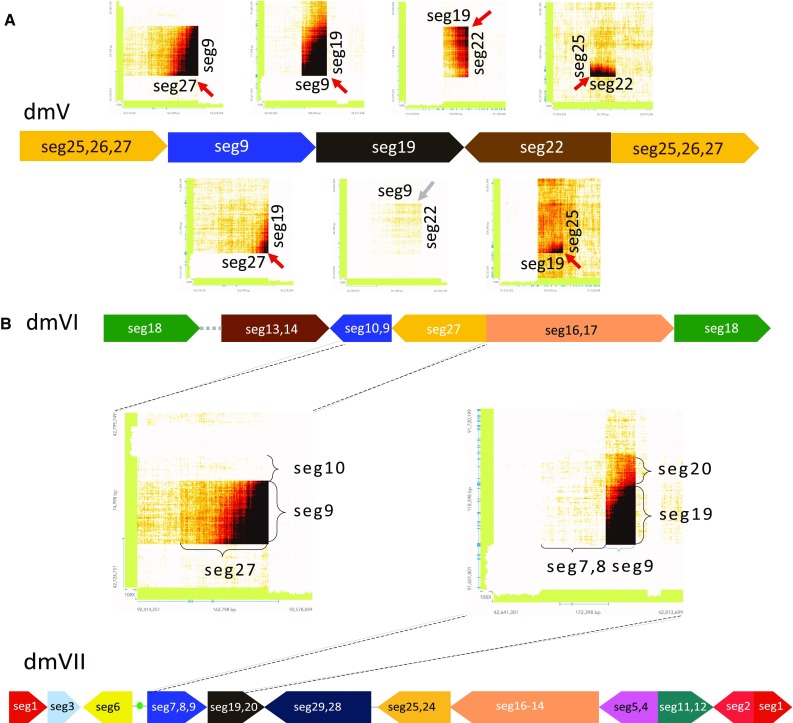
Linked-reads data validation for dmV, dmVI, and dmVII in the relapse sample of SJHGG019. **a** Validation of dmV. The heat maps above dmV represent barcode sharing of the linked-reads between two immediately joined segments, and the heat maps below dmV represent barcode sharing of the linked-reads between two segments spaced by another segment. The red arrows represent the SVs validated by barcode sharing of linked-reads with strong evidence. The gray arrows represent the SV validated by barcode sharing of linked-reads with less strong evidence due to the long distance in between. The scale of the segments’ lengths is slightly adjusted to accommodate all the heat maps. **b** Invalidation of dmVI and dmVII. The heat maps represent barcode sharing of the linked-reads between two immediately joined segments. While seg27 is indeed linked with 5′ of seg9 in dmVI, it stops at 3′ of seg9 and does not extend to seg10 as predicted in the structure. Similarly, while seg19 is indeed linked with 3′ of seg9 in dmVII, it stops at 5′ of seg9 and does not extend to seg8 as predicted in the structure

To construct circular structures that likely represent double minutes, we applied a graph search algorithm. In total, we constructed seven cyclic graphs: dmI, dmII, dmIII, dmIV, dmV, dmVI, and dmVII. Because the average length of the Chromium long molecules for this sample is 31.5 kb, we were able to not only validate the SVs between any two directly linked segments, but also validate whether two distant segments are actually on the same molecule when they are predicted to be on the same double minutes. For example, in dmV did we find that strong barcode sharing is not only present for all the SVs identified (Fig. 2a), but also able to validate that seg27 and seg19 are linked (Fig. 2a) because seg9 is about 27.1 kb (Suppl. Figures 2–4, Online Resource 1), which is shorter than 31.5 kb. Similarly, because seg22 is about 16 kb (Suppl. Figures 2–4, Online Resource 1), we were able to validate that seg19 and seg25 are linked (Fig. 2a). In contrast, because seg19 is about 39 kb (Suppl. Figures 2–4, Online Resource 1), the signal that seg9 and seg22 are on the same molecule is much weaker than the previous two (Fig. 2a). Notably, using the Chromium linked-reads data, we were also able to invalidate two cyclic graphs, i.e., dmVI and dmVII (Fig. 2b). Specifically, in dmVI, seg27 was predicted to connect with seg9–10, but the linked-reads data show that seg27 is indeed connected to seg9 which does not, however, extend to seg10; therefore, dmVI is a false positive (Fig. 2b). Similarly, in dmVII, seg19 is predicted to be connected to seg7–9, but the linked-reads data show that seg19 is only connected to seg9 and does not extend to seg8 or seg7; therefore, dmVII is also a false positive (Fig. 2b).

For the five validated cyclic graphs, their lengths range from 403 kb (dmV) to 974 kb (dmII) (Suppl. Figure 4, Online Resource 1). The number of SVs in them ranges from 2 (dmIV) to 8 (dmI) (Suppl. Figure 3, Online Resource 1). While some segments are unique to one double minutes, some segments are present in multiple double minutes. For example, while seg28 and seg29 are only present in dmII, seg9 is present in all of them except for dmIII (Suppl. Figures 2–4, Online Resource 1). To confirm that these identified circular structures are double minutes, we did tri-color interphase fluorescence in situ hybridization (FISH) analysis with probes to three amplified oncogenes: *MYC*, *EGFR*, and *CDK6*. The results showed that these three genes are highly amplified and dispersed in the nuclei (Fig. 1b), suggesting that they are amplified on double minutes.

### Copy number and oncogene expression of the double minutes

**Fig. 3:**
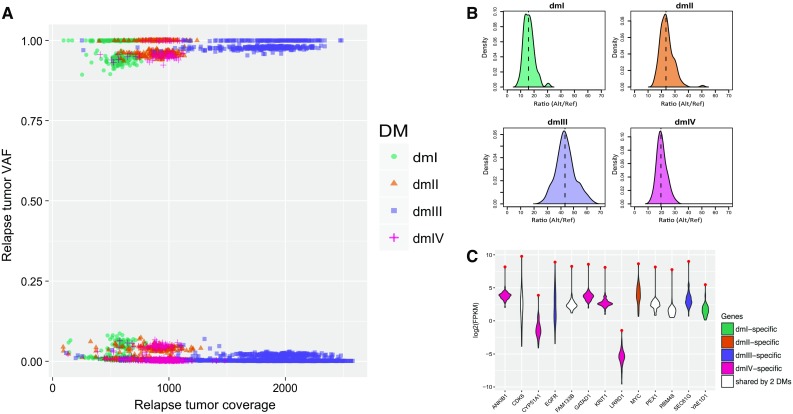
Copy number estimation of the double minutes and expression analysis of the genes carried by them in the relapse sample of SJHGG019. **a** Variant allele frequency (VAF) vs. coverage for SNVs in segments specific to dmI, dmII, dmIII, and dmIV. **b** Distribution of the ratio of alternative allele reads count versus reference allele reads count of the germline SNVs on the upper half of panel A for dmI, dmII, dmIII, and dmIV. The vertical dashed line represents the median of the distribution, i.e., the estimated copy number of the double minute per cell. **c** Gene expression (characterized by log2FPKM) comparison between the relapse sample of SJHGG019 and the other non-brainstem pediatric HGG samples for genes carried by one or two identified double minutes. Each violin plot represents the distribution of gene expression levels across the samples, and the red dot represents the expression level of SJHGG019 relapse sample

Among the five identified double minutes in the relapse tumor, their copy numbers are likely to vary greatly as evidenced by the distinct coverage difference of the segments (Suppl. Figure 2, Online Resource 1) as well as the wildly different number of soft-clipped reads supporting the SVs in each double minutes (Suppl. Figure 3, Online Resource 1). Because segment coverage and number of SV-supporting soft-clipped reads can be affected by many factors such as local GC content, DNA library quality and sequence complexity, we sought to determine double minute abundance by analyzing VAF and coverage of the SNVs on them. We focused on segments unique to a double minutes only. Each double minute includes some unique segments except for dmV. For these unique segments, we extracted all the high-quality germline and somatic SNVs on them and examined the relationship of the VAF against coverage for each double minute (Suppl. Table 2, Online Resource 2). As shown in Fig. 3a, many SNVs have VAF slightly above 0 or slightly below 1. This is expected because these SNVs are located on genomic regions that were highly amplified through double minutes, and depending on whether the amplified segments harbor reference or alternative alleles at the SNVs their VAFs would drift from 0.5 to 0 or 1. Figure 3a also shows a negative correlation between the coverage and VAF’s distance to 0 or 1 among the four double minutes. Specifically, dmIII has the highest coverage and its VAFs are closest to 0 or 1; dmI has the lowest coverage and its VAFs are the farthest from 0 or 1; dmII and dmIV have intermediate and similar coverage and VAFs.

To quantify the copy number of each double minutes, we used germline SNVs whose alternative alleles were amplified by the double minutes, i.e., the upper half of the SNVs in Fig. 3a, because they represent preexisting variants with VAF as 0.5. The approximate copy number of each double minutes relative to one set of normal chromosomes (i.e., one tumor cell) can, therefore, be estimated by the distribution of the ratio of alternative allele reads vs reference allele reads of these germline SNVs (Fig. 3b). Using median of the distribution, we estimated that on average per tumor cell, there are approximately 16 copies of dmI, 23 copies of dmII, 43 copies of dmIII, and 20 copies of dmIV. For dmV, there exists no segment unique to dmV, but seg22 and seg26 are shared by dmIV and dmV (Suppl. Figure 2, Online Resource (1). Using the germline SNVs with alternative alleles amplified, we estimated that the copy number for these two segments is about 33; thus, the copy number of dmV is 13 (i.e., 33–20). Similarly, seg19, seg25, and seg27 are shared by dmII, dmIV and dmV (Suppl. Figure 2, Online Resource 1). Using the same approach, we estimated that the copy number for these three segments is about 57, which makes the copy number of dmV 14 (57–23–20). Therefore, using the germline SNVs from the segments shared by different sets of double minutes, the estimate of copy number of dmV is consistent.

We next investigated the genes on the identified double minutes and their expression level. Each of the five double minutes contains one or more protein-coding genes including oncogenes like *EGFR* (Suppl. Table 1, Online Resource 2). For example, dmII contains *MYC*, dmIII contains *EGFR*, and both dmIV and dmV contain *CDK6* (Suppl. Figure 4, Online Resource 1). We hypothesize that these highly amplified genes have significantly elevated expression level compared to the same genes in other HGG samples. To test this hypothesis, we compared the RNA-seq data (FPKM) of all the double minute-amplified genes in the relapse sample of SJHGG019 with those in 49 other pediatric non-brainstem HGG samples [36], which do not show any signs of amplicons on chromosome 7 or chromosome 8. We find that although different genes show stark difference in their expression level within the SJHGG019 sample, every one of them has the highest expression level among the 50 samples (Fig. 3c). We used z-score to measure the magnitude of expression level increase for genes specific to a double minute. However, we found no clear linear relationship between the copy number of genes and the magnitude of expression level increase.

### The rise and fall of double minutes from diagnosis to relapse

In order to understand the evolutionary dynamics of double minutes, we constructed the structures of double minutes in the diagnosis sample from the same patient. Out of the 372 segments identified by CONSERTING [4], 44 of them have log_2_R > 3 and fall on the right tail of log_2_R distribution (Suppl. Figure 1, Online Resource 1). All of the 44 segments are on Chromosome 7 (Suppl. Figures 2–4, Online Resource 1). Using the same approach that focuses on the reads around segment boundaries, we identified 18 SVs supported by soft-clipped reads, two SVs supported by discordant reads, and four SVs supported by bridging discordant reads. All the identified SVs are confirmed by Chromium linked-reads data (Suppl. Figure 5, Online Resource 1). Moreover, three additional SVs were recovered by the linked-reads data (Suppl. Figures 3 and 5, Online Resource 1). Scanning a network comprising the 44 segments, 27 SVs and 33 pairs of adjacent segments, we identified 52 cyclic graphs, among which 33 were invalidated by Chromium linked-reads data. For the rest of the identified circular structures, we chose five structures that cover all of the 44 segments and 27 SVs and reflect the copy number difference among the segments. We named them dm1, dm2, dm3, dm4, and dm5 (Fig. 1a, Suppl. Figures 2–4, Online Resource 1). For these five double minutes, their length ranges from 640 kb (dm5) to 966 kb (dm4) (Suppl. Figure 4, Online Resource 1). The number of SVs involved in each double minutes ranges from one (dm5) to 16 (dm3) (Suppl. Figure 3, Online Resource 1). Like the double minutes identified in the relapse tumor, in the diagnosis tumor some segments are unique to one double minutes (e.g., segments 23 and 25), and some segments are present in multiple double minutes (e.g., segments 2 and 21, Suppl. Figure 2, Online Resource 1). We also did tri-color interphase FISH analysis with probes to *MYC*, *EGFR*, and *CDK6*. The results suggest that *EGFR* and *CDK6* are highly amplified on different double minutes, and that *MYC* is not amplified, consistent with our constructed double minute structures (Fig. 1b, Suppl. Figure 4, Online Resource 1). Among the five diagnosis double minutes, dm1 has the same structure as dmIII in the relapse tumor except that dm1 carries *EGFRvIII* as evidenced by the deletion of seg14, which leads to *EGFR* exon 2–7 deletion. Further scrutiny of dm1 structure reveals that there is still a portion of dm1 carrying wild-type *EGFR* (*wtEGFR*), suggesting that dmIII in the relapse tumor is likely inherited from dm1 in the diagnosis tumor (see the next section for detailed analysis).

Whether the other four relapse double minutes were generated at relapse or inherited from the diagnosis tumor is unknown. To investigate this, we searched for the SVs present in the relapse double minutes in the diagnosis sample. If all the SVs in a relapse double minutes can be found in the diagnosis sample, then it would suggest that this relapse double minutes is likely to be present in the diagnosis sample. We applied the same approach to search for SVs between the boundaries of the relapse sample’s CNA segments in the diagnosis tumor. In addition to dmIII, we found evidence of other two relapse double minutes present in the diagnosis sample: dmI and dmIV. For dmI, all the SVs were identified in the diagnosis sample, but the number of reads supporting each SV is extremely low (Suppl. Figure 3, Online Resource 1). For example, three SVs that have hundreds of soft-clipped reads support in the relapse sample have only one soft-clipped read support in the diagnosis sample. Similarly, three other SVs that have hundreds of soft-clipped reads support in the relapse sample have only one or two discordant read pairs support and no soft-clipped read support. For dmIV, the two SVs that each has roughly 1000 soft-clipped reads support in the relapse sample have four and two soft-clipped reads support in the diagnosis sample. In contrast, five out of the six SVs in dmII were not found in the diagnosis sample (not counting the two SVs shared with dmIV); and none of the four SVs in dmV was found in the diagnosis sample (not counting the two SVs shared with dmIV). Therefore, our analysis suggests that only dmII and dmV were likely to have formed at relapse, yet dmI and dmIV were present as minor clones in the diagnosis tumor and expanded to be major clones at the time of relapse.

Applying the same principle, we checked whether the five diagnosis double minutes were present in the relapse sample. We did not find any reads supporting any of the SVs in the diagnosis double minutes except for the shared *EGFR*-carrying one (Suppl. Figure 4, Online Resource 1), suggesting that all the major double minutes formed before or at the time of diagnosis have been eliminated by treatment except for the one bearing *EGFR*.

Because the germline blood sample was collected at remission (2 months after the treatment), we also checked whether any of the SVs spanning the breakpoints of the nine double minutes identified in the two tumor samples were present in the matched germline DNA extracted from white blood cells (WBC). But we found no reads evidence supporting any of these SVs in the WBC DNA. It would be of interest for future investigation if any trace amount of cell free DNA fragments unique to the double minutes can be detected in plasma or serum as potential early diagnosis markers.

### dm1 evolution

**Fig. 4:**
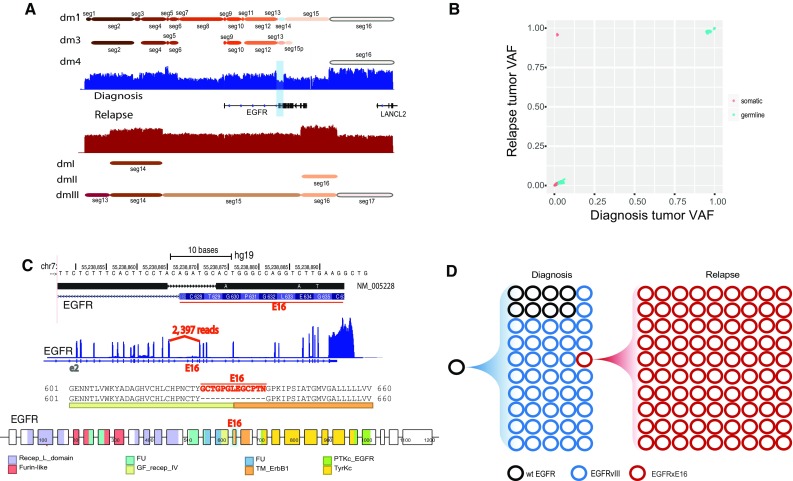
Characterization and evolution of the shared double minutes between the diagnosis and relapse samples of SJHGG019. **a** Highly amplified segments around *EGFR* are involved in multiple double minutes in both samples. Seg14 deleted in dm1 results in *EGFRvIII*. **b** Variant allele frequency comparison of the SNVs shared by dm1-specific segments in the diagnosis sample and dmIII-specific segments in the relapse sample. On the upper left corner are four somatic SNVs with low frequency (0.02) in the diagnosis sample and high frequency (0.96) in the relapse sample. **c** Among the four somatic SNVs, three are located close to each other on exon 16 of *EGFR*. Along with these three SNVs is an eight base pair deletion that disrupts the intron15/exon16 splicing acceptor site, leading to skipping of exon 16 in the *EGFR* RNA transcript, indicated by the missing coverage of exon16 in RNA-seq coverage track and large number of supporting reads for the novel splice junction. Consequently, the skipping of exon 16 produces an *EGFR* protein product with an in-frame deletion of 13-amino acids (GCTGPGLEGCPTN), which partially code for two protein domains: growth factor receptor domain IV (GF_recep_IV) and transmembrane ERBB1 like domain (TM_ErbB1). **d** Evolution of the *EGFR*-containing double minutes from diagnosis (dm1) to relapse (dmIII). Black circles represent double minutes carrying wild-type *EGFR* (*wtEGFR*), blue circles represent double minutes carrying *EGFRvIII*, and red circles represent double minutes carrying *EGFR* with exon 16 deletion in RNA transcript (*EGFRxE16*). The counts of circles in different colors reflect the relative abundance of each type of *EGFR* as estimated from sequencing data

As mentioned in the previous section, dmIII in the relapse tumor shares the same structure as dm1 in the diagnosis tumor. In this section, we aimed to investigate the evolution of the *EGFR*-carrying double minutes from the diagnosis tumor to the relapse tumor. Since some segments involved in dm1 or dmIII are also part of other double minutes structures (Fig. 4a and Suppl. Figure 2, Online Resource 1), we extracted the high-quality diagnosis SNVs on dm1-specific segments (seg1, seg3, seg7, seg8, seg11, seg15, seg18, and seg20) and the high-quality relapse SNVs on dmIII-specific segments (seg13, seg15, seg17, and seg18) and intersected them based on their genomic locations. We identified 227 such shared SNVs between the two samples. The vast majority of them show similar VAFs close to 0 or 1 in the two samples (Fig. 4b), consistent with the fact that these SNVs are highly amplified and that dmIII is inherited from dm1. Intriguingly, there are four somatic SNVs showing low VAFs (around 0.02) in the diagnosis sample but high VAFs (around 0.96) in the relapse sample (Fig. 4b), suggesting clonal evolution of the double minutes. Among the four SNVs, one is located about 8700 bp upstream of *EGFR*, and the other three are all located close to each other on exon 16 of *EGFR* (Fig. 4c). More importantly, by examining the reads carrying these three somatic SNVs we identified a co-occurring somatic 8-bp deletion (ACAGATGC, on the same haplotype) that disrupts the intron15/exon16 splicing acceptor site, leading to skipping of exon 16 in the *EGFR* RNA transcript, as indicated by the missing coverage of exon16 in RNA-seq coverage track and 2793 reads spanning exon 15 and exon 17 junctions (Fig. 4c). Consequently, the exon 16 skipping produces an *EGFR* protein product with an in-frame deletion of 13-amino acids (GCTGPGLEGCPTN), which partially code for two protein domains: growth factor receptor domain IV and transmembrane ERBB1 like domain (Fig. 4c). For convenience, we termed the observed exon16 skipping of the *EGFR* RNA transcript as *EGFRxE16*.

So far we determined that a small proportion (2%) of dm1 carry *EGFRxE16* and but it is unclear what percentage of dm1 carry *EGFRvIII* or *wtEGFR*. *EGFRvIII* in the diagnosis sample is caused by the deletion of seg14 (Fig. 4a and Suppl. Figure 3, Online Resource 1), and seg14 is used by dm3 as evidenced by soft-clipped reads and Chromium linked-reads (Suppl. Figure 6, Online Resource 1). Therefore, we reasoned that evidence showing seg14 is also involved in dm1 would show that *wtEGFR* is carried by dm1. We first compared the average coverage of seg14 with those of dm3-specific segments, i.e., seg24, seg25, seg26, and seg27. The log_2_R of seg14 is 4.145, higher than those of seg24 (3.468), seg25 (3.501), seg26 (3.434), and seg27 (3.489), suggesting that seg14 is amplified at somewhere else other than dm3. Since seg14 is not involved in dm2, dm4, and dm5, it is most likely to be present in dm1, leading to *wtEGFR*. To quantify the proportion of *wtEGFR*-carrying dm1, we first estimated the copy number of dm1 (all versions together) and dm3 using the germline SNVs in the dm1- and dm3-specific segments using the same approach mentioned in previous section: they are estimated to be 24 and 15, separately. We then estimated the copy number of seg14 (shared by a fraction of dm1 and dm3) to be 19. Therefore, the proportion of *wtEGFR*-carrying dm1 is estimated to be (19–15)/24 = 16.7%. Based on these estimates and the previously estimated copy number of dmIII in the relapse sample, we constructed the evolutionary history of the *EGFR*-carrying double minutes from diagnosis to relapse (Fig. 4d). While the treatment effectively eliminated both *wtEGFR* and *EGFRvIII*, it failed to eliminate *EGFRxE16* which ultimately expanded in the relapse sample.

Interestingly, we noticed that while *EGFRvIII* was present predominantly in adult GBM and low-grade gliomas, *EGFRxE16* isoforms were observed in a wide spectrum of adult cancers, including GBM, LGG, ESCA, HNSC and others (Suppl. Table 3, Online Resource 2). Moreover, this isoform is not present in the RNA-seq of 683 normal brain tissues (in HDBR) or 95 other normal tissues examined, as wells as the EST database (UCSC). Notably, exon 16 codes for a segment of the extracellular domain close to the dimerization of the *EGFR* receptors, a process important for auto-inhibition of *EGFR* signaling pathway. Interestingly, an *SEC61G*-*EGFR* fusion gene that consists of the first exon of *SEC61G* and exon 16 skipping versions of *EGFR* (MF434546: exon14, 15, 17–28; and MF434547: exon15, 17–28) was reported in pediatric ependymomas recently [23]. Taken together, the *EGFR* isoform skipping exon 16 deserves further functional characterization in the future.

### Amplicons in other paired diagnosis and relapse GBM samples

To understand whether the observed pattern of double minute evolution from the diagnosis tumor to the relapse tumor is unique to SJHGG019 or more commonplace, we examined the highly amplified segments (log_2_R > 3) in other sets of paired diagnosis and relapse samples from TCGA adult (GBM) patients. Among the 37 TCGA GBM cases, nine cases have WGS data available for both diagnosis tumor and relapse tumor. Among the nine pairs of WGS samples, four pairs show striking amplicon amplifications: TCGA-06-0125, TCGA-06-0152, TCGA-06-0211, and TCGA-14-1402. We, therefore, analyzed their amplicons and the associated SVs in these eight samples. We find that except for TCGA-06-0125 whose diagnosis and relapse sample shares the same one CNA segment and the same one SV (Suppl. Figure 7A, Online Resource 1), all the pairs have largely different CNA profiles and their associated SVs between their diagnosis and relapse samples. For example, TCGA-06-0152 has 18 SVs connecting segments on chromosome 7 and chromosome 12 in the diagnosis sample, but has only 3 SVs connecting segments on chromosome 7 in the relapse sample (Suppl. Figure 7B, Online Resource 1). In addition to TCGA-06-0125 sharing the one SV between the two samples, we also found that TCGA-06-0211 shares three SVs between the 13 SVs identified in the diagnosis sample and the 21 SVs identified in the relapse sample (Suppl. Figure 7C, Online Resource 1), and that TCGA-14-1402 shares three SVs between the three SVs identified in the diagnosis sample and the eight SVs identified in the relapse sample (Suppl. Figure 7D, Online Resource 1).

Like SJHGG019, we also cross-checked the SVs identified in diagnosis or relapse sample in the other sample for each case. This reveals great difference between the four cases. For TCGA-06-0125, the one and only SV is identified in both samples, suggesting that the same DM was maintained at high amount in both diagnosis and relapse samples. For TCGA-06-0152, none of the 18 SVs identified in the diagnosis sample was found in the relapse sample, and none of the three SVs identified in the relapse samples was found in the diagnosis sample. For TCGA-06-0211, 11 out of the 13 SVs identified in the diagnosis sample was found in the relapse sample, but most of them have less than five supporting reads, compared to hundreds of supporting reads in the diagnosis sample; all the 21 SVs identified in the relapse samples were found in the diagnosis sample with moderate amount of supporting reads for most SVs. For TCGA-14-1402, except for the three shared SVs, no other SVs identified in the relapse sample were found in the diagnosis sample. The other SVs in the relapse sample could be secondary genomic rearrangement on the formed double minutes. While these eight adult GBM samples were previously analyzed for eccDNA [5], our results highlight the maintenance, disappearance, novel formation, and abundance change of double minutes longitudinally.

## Discussion

EccDNA have been detected in both normal and neoplastic tissues in humans as well as other species [18, 24, 25]. In tumor cells, their sizes are much larger than those found in normal tissues and they usually contain oncogenes or proto-oncogenes [20, 28]. Compared to the relatively long history of experimental observation of double minutes in tumor cells, efforts to characterize eccDNA structures have just started and can be roughly divided into three categories: (1) reconstructing eccDNA directly using WGS short-reads data [9, 22, 32], (2) purification of eccDNA followed by high-throughput sequencing [18, 25], and (3) long-reads sequencing followed by de novo assembly [5]. Among the publicly available tools [9, 32], we applied AmpliconArchitect on our relapse sample of SJHGG019 to compare with our results [32]. Under its “EXPLORE” mode, the tool successfully identified three circular structures, corresponding to our dmII, dmIV, and dmV. It did not report dmI because dmI contains a SV that was only uncovered by the bridging discordant reads and AmpliconArchitect did not use this information. It also did not report dmIII because dmIII contains a SV that was only recoverable by the Chromium linked-reads. In addition, AmpliconArchitect did not report exact break points for the SVs because it uses discordant reads information but not soft-clipped reads information. We, therefore, employed our own approach that integrates multiple lines of evidence to construct double minute structures in both samples of SJHGG019 to study the evolution of double minutes.

It has been recognized that double minutes can be inserted back into linear chromosomes to form homogeneously staining regions (HSRs) [28], but computationally it is difficult to distinguish eccDNA and HSRs. Our FISH image shows that in the diagnostic sample *EGFR* and *CDK6* were amplified as eccDNA, not HSR. In the sample from relapsed tumor, there exist some large signals for *EGFR*, *CDK6*, and *MYC* along with the widely distributed smaller signals consistent with eccDNA; therefore, we cannot rule out the possibility that HSR may co-exist with double minutes at this time. However, whether the amplicon is a linear or circular form will not affect any of our results including their structure, segment orientation, abundance, and evolution, as well as the secondary somatic mutations.

Our results demonstrated that while *MYC* amplification was only observed in the relapse sample but not in the diagnosis sample of SJHGG019, *EGFR* and *CDK6* amplification were maintained in both of the samples. Importantly, while *EGFR* was amplified by the same double minutes in both samples, *CDK6* was amplified by different double minute structures in the diagnosis sample (dm4 and dm5) compared to the relapse sample (dmIV and dmV, Suppl. Figures 2–4, Online Resource 1). Similarly, in TCGA-06-0152 and TCGA-06-0211, although *EGFR* region was highly amplified in both diagnosis and relapse samples, the segments’ boundaries and their associated SVs suggest that *EGFR* was likely amplified by different double minute structures between the two samples (Suppl. Figure 7, Online Resource 1). It suggests that longitudinal maintenance of oncogene amplification does not necessarily imply the longitudinal maintenance of double minute structures.

We found dynamic copy number shift of different double minutes from diagnosis to relapse tumors. Specifically, for SJHGG019 one relapse double minutes shares the same structure with a diagnosis double minutes, and two other relapse double minutes can also be found in the diagnosis sample but in a trace amount; in contrast, none of the other four diagnosis double minutes can be found in the relapse sample. The longitudinal dynamics were further confirmed in another four pairs of diagnosis/relapse samples of GBM patients. For example, in TCGA-06-0152, none of the relapse amplicon-associated SVs can be found in the diagnosis sample and none of the diagnosis amplicon-associated SVs can be found in the relapse sample. In contrast, in TCGA-06-0211, all the relapse amplicon-associated SVs can be found in the diagnosis sample with moderate amount of reads support and all the diagnosis amplicon-associated SVs can be found in the relapse sample with trace amount of reads support. It indicates that tumor evolution involves a dynamically changing composition of double minutes in addition to clonal evolution in the representation of chromosomal mutations. This evolution can be driven by selective advantage of newly acquired mutations, changes in the tumor microenvironment, or response to therapy, much like bacteria under antibiotics selection. Admittedly, we cannot rule out the possibility that samples biopsied at diagnosis and relapse include topographically segregated sub-clones due to regional tumor heterogeneity. For example, although some relapse double minutes were not found in the paired diagnosis sample for SJHGG019 and TCGA-06-0152, it is possible that the diagnosis samples did not contain any cells that harbor these relapse double minutes because of different sampling regions. Similarly, we did not find evidence for most of the diagnosis double minutes in the paired relapse sample for SJHGG019 and TCGA-06-0152, which could be because treatment such as surgical removal of cancer cells, or response to radiation and/or chemotherapy eliminated these double minutes. Patient SJHGG019 was treated with the *EGFR* inhibitor erlotinib. Although *EGFRvIII* is somewhat inhibited by erlotinib, this GBM-associated mutation is less sensitive to erlotinib than the *EGFR* kinase domain mutations found in lung cancer [33]. *WtEGFR* also plays a critical oncogenic role in GBM, contributing to tumor cell invasion [29], and heterogeneous co-expression of wild type and mutant *EGFR* contributes to drug resistance in GBM [37]. Emergence of *EGFR* mutations following radiation and temozolomide treatment of GBM indicates that dysregulated *EGFR* signaling can contribute to late expansion of tumor [35]. Future experiments using in vitro cell lines or in vivo patient-derived xenograft models would greatly advance our understanding of the evolution of double minutes under drug treatment and how they segregate to descendant tumor cells.

**Fig. 5:**
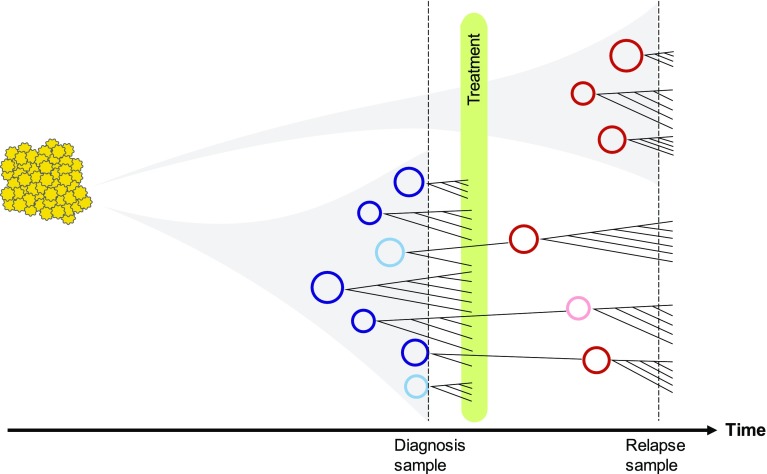
A schematic diagram illustrating the evolutionary trajectory of double minutes. The timeline at the bottom shows the time at diagnosis, treatment and relapse. Each circle represents a double minutes with unique structure. Circles in blue and maroon represent highly amplified double minutes in the diagnosis and relapse samples; circles in light blue and light maroon represent double minutes with low abundance in diagnosis and relapse samples. Every double minutes is mutable and can acquire new somatic SNVs, INDELs, and SVs which are then subject to selection. While some diagnosis double minutes may be eliminated by treatment, some may survive and expand. In addition, new double minutes can form after the treatment

We also found evidence that secondary somatic mutations including point mutations, INDELs, and rearrangements can occur on the double minutes after they are formed. Because one cell may contain multiple copies of a double minute, each one of these copies in just one cell could gain different mutations in the same genomic region in one round of replication, which leads to accelerated evolution of double minutes. Therefore, the evolution of double minutes is independent of and faster than that of regular linear chromosomes. We propose an evolutionary model that simultaneously involves a branching model for double minute formation and a selection model for secondary mutations on the formed double minutes (Fig. 5). Based on our observations that new double minutes were found in both diagnosis and relapse samples, we infer that new double minutes can arise any time before diagnosis to the time of relapse and beyond. Once a double minute is formed, secondary mutations can occur, leading to competition between different versions of this double minute and different versions of other double minutes. The final presence and quantity of different double minutes depend on how much they facilitate tumor growth or confer drug resistance.

As mentioned in the results, our current approach cannot detect a SV if the repetitive or unknown region between two segments (e.g., between seg18 and seg13 in dmIII) is longer than the insert size, which can prevent circular structures from being detected. This can be recovered by long reads sequencing such as Chromium linked-read sequencing used in our study. Although the linked-read data can be used to validate or invalidate individual SVs in a predicted double minutes, they do not necessarily validate the entire double minute structure because the average length of the linked-read molecule is ~ 30 kb. This limitation can be potentially mediated by other long read sequencing technologies like PacBio or Nanopore, followed by long read assembly. Notably, although some cyclic graphs could be computationally valid, several cyclic graphs predicted here have been shown to be false positives by our Chromium data. Therefore, we recommend to validate short-read predicted amplicon structures with orthogonal long read sequencing in future double minute studies if the amplicon structures are critical to understand the biological or clinical questions.

In summary, we performed in-depth analyses of the populations of different double minutes in the paired tumors in multiple GBM patients. For the pediatric patient, not only did we determine the exact breakpoints, the segments and their orders/orientations in each double minutes, we also determined the copy numbers as well as the dynamics of each double minute population at two time points. For the first time, we also examined the secondary somatic mutations on the double minutes and their influence on double minute evolution. We proposed a model to summarize the dynamic trajectories of double minute evolution.

## Electronic supplementary material

Below is the link to the electronic supplementary material.

Supplementary material 1 (DOCX 18804 kb)

Supplementary material 2 (XLSX 40 kb)

Supplementary material 3 (XLSX 1050 kb)

Supplementary material 4 (XLSX 22 kb)

## Data Availability

Illumina and Chromium sequencing data of the tumor samples from the HGG patient have been made available at European Bioinformatics Institute under accession EGAS00001000192, and EGAS00001003212, respectively.
