# Supplementary material for: Structure and evolution of double minutes in diagnosis and relapse brain tumors
Source: Acta Neuropathol. 2018 Sep 28;137(1):123–37. doi: 10.1007/s00401-018-1912-1 (PMC6338707; doi:10.1007/s00401-018-1912-1)
Supplement: Supplementary file 1 — Supplementary material 1 (DOCX 18804 kb) [file 401_2018_1912_MOESM1_ESM.docx]

*Supplementary figures and tables*

**Structure and evolution of double minutes in diagnosis and relapse brain tumors**

Ke Xu^1^, Liang Ding^1^, Ti-Cheng Chang^1^, Ying Shao^1^, Jason Chiang^2^, Heather Mulder^1^, Shuoguo Wang^1^, Tim I. Shaw^1^, Ji Wen^2^, Laura Hover^3^, Clay McLeod^1^, Yong-Dong Wang^1^, John Easton^1^, Michael Rusch^1^, James Dalton^2^, James R. Downing^2^, David W. Ellison^2^*, Jinghui Zhang^1^*, Suzanne J. Baker^3^*, Gang Wu^1^*

Department of Computational Biology^1,^ Department of Pathology^2^, Department of Developmental Neurobiology^3,^

St. Jude Children’s Research Hospital 262 Danny Thomas Pl, Memphis, TN 38105 USA

*Corresponding authors

David W. Ellison: [david.ellison@stjude.org](mailto:david.ellison@stjude.org)

Jinghui Zhang: [jinghui.zhang@stjude.org](mailto:jinghui.zhang@stjude.org)

Suzanne J. Baker: [suzanne.baker@stjude.org](mailto:suzanne.baker@stjude.org)

Gang Wu: [gang.wu@stjude.org](mailto:gang.wu@stjude.org)

**Supplementary Figures**


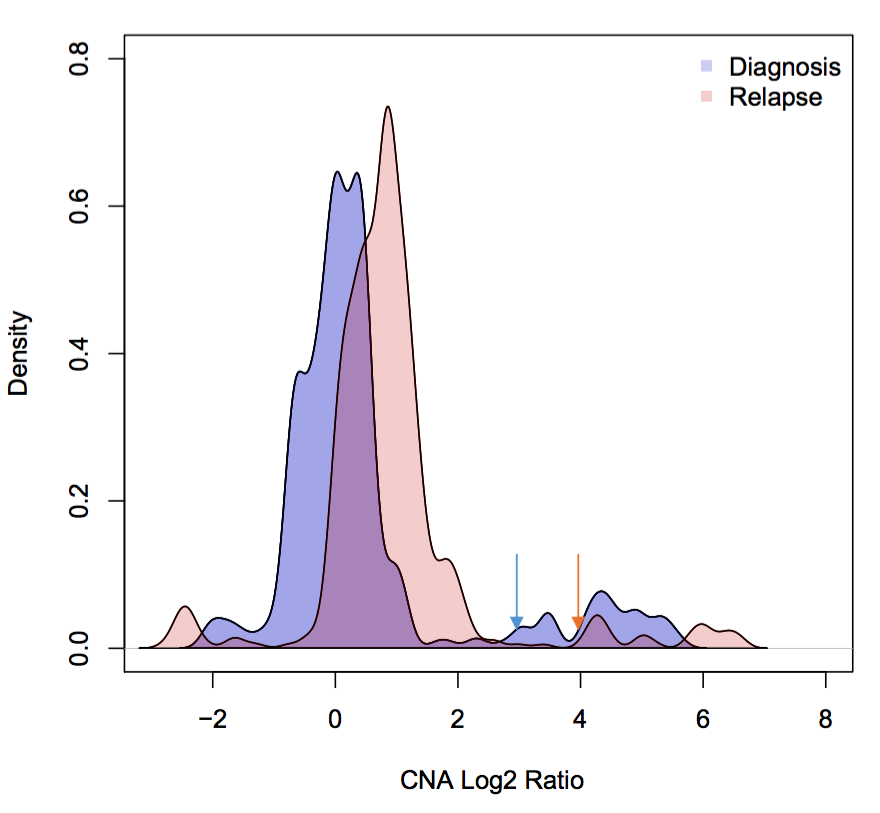


**Supplementary Figure 1. Log_2_R (between tumor sample and its matching germline sample) distribution of all the segments generated by CONSERTING for the diagnosis sample and relapse sample of SJHGG019.** The blue and red arrows indicate arbitrary cutoffs for highly amplified CNA segments in the diagnosis sample and relapse sample, respectively.

**Supplementary Figure 2. Distribution of the highly amplified CNA segments in the predicted double minute structures in the diagnosis sample and relapse sample of SJHGG019.** The middle track shows the segments and their coverage in the diagnosis sample (blue) and relapse sample (maroon). The range of y-axis (coverage) is [0, 4000]. The tracks below the coverage tracks show the participating segments in each constructed double minute structure in the relapse sample. The tracks above the coverage tracks are the participating segments in each constructed double minute structure in the diagnosis sample. For each sample, each unique segment is in different color and adjacent segments have gradient colors.

**Supplementary Figure 3. The predicted structures of double minutes identified in the diagnosis sample and the relapse sample of SJHGG019.** For each double minutes, the first and the last segments are the same and have the same orientation, representing its circular nature. For each sample, each unique segment is in a different color as shown in Supplementary Figure 2. Segments directly linked to each other means SVs supported by soft-clipped reads. Segments linked by a solid grey line means SVs supported by discordant reads but not soft-clipped reads. Segments linked by a solid grey line plus a green dot means segments connected by a bridging segment. Segments linked by a dotted grey line means SVs identified by Chromium linked-reads only. Exact break point positions are shown below the segment boundaries if there exists soft-clipped reads support, otherwise approximate break point positions are shown (with notation “*”). The numbers without bracket above and below each SV represent the numbers of supporting soft-clipped reads in the diagnosis sample and the relapse sample, respectively. Similarly, the numbers in round brackets represent the numbers of discordant reads supporting the SVs, and the numbers in square brackets represent the numbers of discordant reads supporting the bridging segment. In panel B, ^h^ means only half of the supporting reads are counted. The light blue lines connecting SVs between different double minutes represent the same SVs shared by different double minutes.

**Supplementary Figure 4. Circular visualization of the predicted double minute structures in the diagnosis sample and the relapse sample of SJHGG019.** The size of each double minutes and the segments is roughly in proportion with its actual predicted size. *CDK6*, *EGFR*, and *MYC* genes are marked at their approximate genomic locations.

**Supplementary Figure 5. Chromium linked-reads sequencing validation for all the SVs except for the soft-clipped reads-supported ones in the predicted double minutes in the relapse sample (A, B, C) and the diagnosis sample (D-L) of SJHGG019.** The soft-clipped reads-supported SVs are not shown here because the evidence from conventional short-reads sequencing is already strong.

**Supplementary Figure 6. Chromium linked-reads sequencing validation for a SV in dm3 of the diagnosis sample of SJHGG019.** The barcode sharing heatmap shows that seg24 is linked to seg15^head^ and extends to seg14, suggesting that seg14 is indeed present in dm3.

**Supplementary Figure 7. CIRCOS plots showing the highly amplified genomic segments and their associated SVs in the paired diagnosis and relapse samples in four TCGA GBM patients: TCGA-06-0125 (A), TCGA-06-0152 (B), TCGA-06-0211 (C), and TCGA-14-1402 (D).** From the most inner circle to the most outer circle represent the highly amplified genomic segments, sequencing coverage of the diagnosis/relapse sample (blue/maroon), sequencing coverage of the relapse/diagnosis sample (maroon/blue), and genomic coordinates (x 1,000 kb) of the chromosomes. SVs supported by different types of evidence are shown by connecting lines with different colors. Genes of interest are marked at their approximate genomic locations.

**Supplementary Tables**

**Supplementary Table 1** Genomic coordinates of the highly amplified CNA segments and the identified SVs between the segment boundaries in the diagnosis and relapse samples of SJHGG019.

**Supplementary Table 2** Information of the high quality SNVs on segments specific to dmI, dmII, dmIII, or dmIV (data used for Figure 3A).

**Supplementary Table 3** The frequency of *EGFRvIII* and *EGFRxE16* isoforms in 11,094 TCGA adult tumors.
